# Investigating Novel Genes Potentially Involved in Endometrial Adenocarcinoma using Next-Generation Sequencing and Bioinformatic Approaches

**DOI:** 10.7150/ijms.38219

**Published:** 2019-09-07

**Authors:** Feng-Hsiang Tang, Wei-An Chang, Eing-Mei Tsai, Ming-Ju Tsai, Po-Lin Kuo

**Affiliations:** 1 Graduate Institute of Clinical Medicine, College of Medicine, Kaohsiung Medical University, Kaohsiung 807, Taiwan; 2 Department of Obstetrics and Gynecology, Kaohsiung Medical University Hospital, Kaohsiung Medical University, Kaohsiung 807, Taiwan; 3 Department of Obstetrics and Gynecology, Kaohsiung Municipal Ta-Tung Hospital, Kaohsiung Medical University, Kaohsiung 807, Taiwan; 4 Division of Pulmonary and Critical Care Medicine, Kaohsiung Medical University Hospital, Kaohsiung 807, Taiwan; 5 Department of Internal Medicine, School of Medicine, College of Medicine, Kaohsiung Medical University, Kaohsiung 807, Taiwan; 6 Graduate Institute of Medicine, College of Medicine, Kaohsiung Medical University, Kaohsiung 807, Taiwan; 7 Department of Respiratory Therapy, College of Medicine, Kaohsiung Medical University, Kaohsiung 807, Taiwan; 8 Institute of Medical Science and Technology, National Sun Yat-Sen University, Kaohsiung 804, Taiwan

**Keywords:** endometrial cancer, papillomavirus, next generation sequencing, bioinformatics, miR-127-5p, miR-218-5p, CSTB, HPGD

## Abstract

Endometrial cancer is one of the most common cancers in women worldwide, affecting more than 300,000 women annually. Dysregulated gene expression, especially those mediated by microRNAs, play important role in the development and progression of cancer. This study aimed to investigate differentially expressed genes in endometrial adenocarcinoma using next generation sequencing (NGS) and bioinformatics. The gene expression profiles and microRNA profiles of endometrial adenocarcinoma (cancer part) and normal endometrial tissue (non-cancer part) were assessed with NGS. We identified 56 significantly dysregulated genes, including 47 upregulated and 9 downregulated genes, in endometrial adenocarcinoma. Most of these genes were associated with defense response, response to stimulus, and immune system process, and further pathway analysis showed that human papillomavirus infection was the most significant pathway in endometrial adenocarcinoma. In addition, these genes were also associated with decreased cell death and survival as well as increased cellular movement. The analyses using Human Protein Atlas, identified 6 genes (*PEG10*, *CLDN1*, *ASS1*, *WNT7A*, *GLDC*, and *RSAD2*) significantly associated with poorer prognosis and 3 genes (*SFN*, *PIGR*, and *CDKN1A*) significantly associated with better prognosis. Combining with the data of microRNA profiles using microRNA target predicting tools, two significantly dysregulated microRNA-mediated gene expression changes in endometrial adenocarcinoma were identified: downregulated hsa-miR-127-5p with upregulated *CSTB* and upregulated hsa-miR-218-5p with downregulated *HPGD*. These findings may contribute important new insights into possible novel diagnostic or therapeutic strategies for endometrial adenocarcinoma.

## Introduction

Cancers of the corpus uteri, primarily from the endometrium, rank as the sixth most common neoplasm in women worldwide. The incidence increased from 290,000 in 2008 to over 380,000 in 2018 1. Estrogen exposure, either endogenous or exogenous, is a major risk factor of endometrial cancer, while endometrial cancer is generally divided into two distinct types, type I (estrogen-related) and type II (non-estrogen-related) 2. As mentioned in a large review, strong evidence suggested that three factors were associated with endometrial cancer: increased body mass index and increased waist‐to‐hip ratio were associated with increased risk, while increased parity reduced the risk of disease 3. The genetic mechanism underlying the pathogenesis of endometrial cancer is not fully understood. In type I endometrial cancer, which account for nearly 80% of endometrial cancer, *PTEN* mutation, *hMLH1* methylation, and *hMSH6* mutation are important in atypical hyperplastic change of normal endometrium. Mutations in *PTEN*, *KRAS*, and *CTNNB1* are associated with malignant change from atypical endometrial hyperplasia to low-grade endometrioid cancer, while *P53* mutation plays an important role in advancing low-grade cancer into high-grade one 4. In type II endometrial cancer, mutations in *P53* and *HER2/neu* are associated with non-endometrioid malignant transformation from normal or atrophic endometrium 4.

Traditionally, patients suffered from endometrial cancer have a favorable treatment outcome if diagnosed in the early stage. The overall five-year survival rate of endometrial cancer is 81%, but is only 17% if distal metastasis occurs 5. The three-year overall survival rate is 96.2% for women without recurrence; however, it is 73.4% for women with vaginal vault recurrence, loco-regional nodal recurrence, or local central pelvic recurrence, and is only 38.1% for those with distal metastases and/or peritoneal carcinomatosis 6. This might be result from the absence of a perfect treatment modality for advanced or recurrent disease currently.

The development of next-generation sequencing (NGS) technologies provides the capability to rapidly sequence exomes, transcriptomes, and genomes at relatively low cost. The application of this technology to catalog the mutational landscapes of tumor exomes, transcriptomes, and genomes has remarkably accelerated the progress in basic and clinical cancer researches 7, making precision medicine possible 8. Individual cancer patients can therefore receive personalized care with the most suitable drugs at the appropriate dose and at the right time 8.

As microRNAs have the ability to repress the expression of protein-coding genes, they might contribute to the pathogenesis of various diseases including cancer 9-13. Functional studies have shown that microRNA dysregulation plays important role in the development and progression of various cancers 9. Some microRNAs may act as either tumor suppressors (miR onco-suppressors) or tumor enhancers (onco-miRs), and anti-cancer treatment with microRNA mimics or molecules targeted at miRNAs are under development. With increasing knowledge of the microRNA-mediated changes in cancer cells, we will have better opportunity to develop a better microRNA-based anti-cancer treatment.

Through identifying novel gene expression signature and microRNA-gene interactions in endometrial adenocarcinoma, we may provide new perspectives for the development of novel diagnostic methods, prognostic predicting tools, and therapeutic strategies of endometrial adenocarcinoma. Therefore, in this study, we would like to identify the differentially expressed gene and the potential regulatory mechanisms through microRNAs in endometrial adenocarcinoma with systematic bioinformatics analysis.

## Materials and methods

### Study design

**Figure 1:**
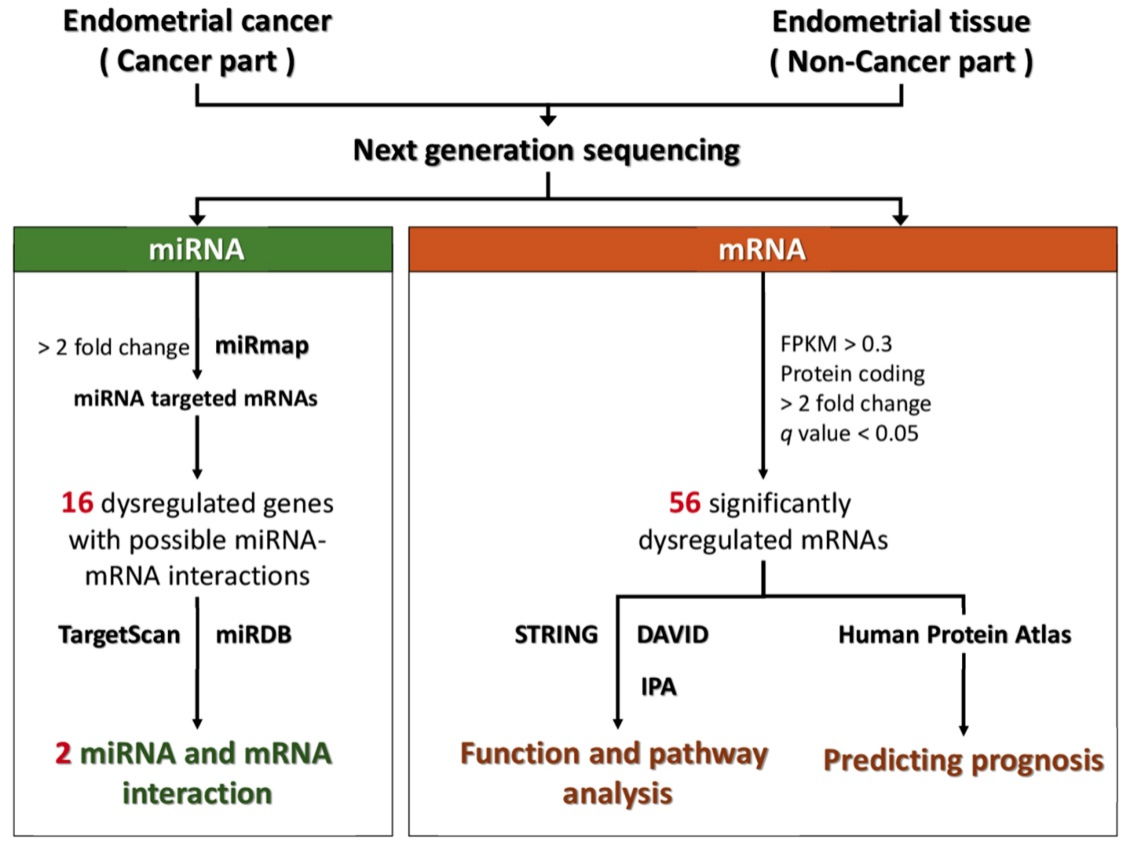
** Flow chart of the study.** Abbreviation: STRING, Search Tool for the Retrieval of Interacting Genes; DAVID, Database for Annotation, Visualization and Integrated Discovery; KEGG, Kyoto Encyclopedia of Genes and Genomes; IPA, Ingenuity^®^ Pathway Analysis.

The flowchart of study design is illustrated in Figure 1. The cancer part and non-cancer part (normal endometrial tissue) were taken from the surgical specimen of a 53-year-old woman with stage Ia endometrial adenocarcinoma cancer after informed consent was obtained. This pair of tissues was sent for NGS to assess the expression profiles of mRNAs and microRNAs. Using bioinformatic tools, including Search Tool for the Retrieval of Interacting Genes (STRING), the Database for Annotation, Visualization and Integrated Discovery (DAVID), and Ingenuity^®^ Pathway Analysis (IPA), the altered functions and pathways related to the dysregulated genes in endometrial cancer were investigated. In addition, the potential targets of the significantly dysregulated microRNAs were predicted with miRmap, TargetScan, and miRDB, and the potential microRNA-mRNA interactions in endometrial cancer were identified.

### NGS for microRNA and mRNA expression profiles

The expression profiles of microRNAs and mRNAs were examined using NGS as in our previous studies 10, 11, 13-16. In brief, total RNA was extracted with Trizol^®^ Reagent (Invitrogen, USA) as per the instruction manual. The purified RNAs were quantified at O.D._260nm_ with a ND-1000 spectrophotometer (Nanodrop Technology, Wilmington, DE, USA) and qualitatively assessed with Bioanalyzer 2100 and RNA 6000 LabChip kit (both from Agilent Technology, Santa Clara, CA, USA). Library preparation and sequencing were performed in Welgene Biotechnology Company (Taipei, Taiwan).

For transcriptome sequencing, the Agilent's SureSelect Strand Specific RNA Library Preparation Kit was used to construct the libraries, followed by AMPure XP Beads size selection. The sequence was directly determined using Illumina's sequencing-by-synthesis (SBS) technology. Sequencing data (FASTQ files) were generated by Welgene's pipeline based on Illumina's base-calling program bcl2fastq v2.2.0. After adaptor clipping and sequence quality trimming with Trimmomatics (Ver. 0.36) 17, alignment of the qualified reads were performed using HISAT2 18, 19, which is a fast and sensitive alignment program for mapping NGS reads to genomes based on hierarchical graph FM index. The genes with low expression levels (< 0.3 fragment per kilobase of transcript per million mapped reads [FPKM]) in any group were excluded. The *p* values were calculated by Cuffdiff with non-grouped samples using the "blind mode”, in which all samples were treated as replicates of a single global "condition" and used to build a model for statistical test 20, 21. The *q* values were the *p* values adjusted with false discovery rate using the method by Benjamini and Hochberg 22. Genes with *q*-value < 0.05 (i.e., -log_10_(*q* value) > 1.3) and > 2-fold changes were considered significantly differentially expressed.

For small RNA sequencing, samples were prepared using Illumina sample preparation kit as per the TruSeq Small RNA Sample Preparation Guide. The 3' and 5' adaptors were ligated to the RNA, and then reverse transcription and PCR amplification were performed. The cDNA constructs were size-fractionated and purified using a 6% polyacrylamide gel electrophoresis and the bands corresponding to the 18-40 nucleotide RNA fragments (140-155 nucleotide in length with both adapters) were extracted. After sequencing on an Illumina (San Diego, CA, USA) instrument (75 bp single-end reads), the data was processed with the Illumina software. After trimming and filtering out low-quality data with Trimmomatics 17 and clipping the 3' adapter sequence and discarding reads shorter than 18 nucleotides with miRDeep2 23, the qualified reads were aligned to the human genome from University of California, Santa Cruz (UCSC). Because microRNAs usually map to few genomic locations, only reads mapped perfectly to the genome ≤5 times were taken. MiRDeep2 is useful for estimating the expression levels of known microRNAs, as well as identifying novel microRNAs. The microRNAs with low levels (<1 normalized read per million (rpm)) in both groups were excluded. The microRNAs with >2 fold change are considered significantly changed.

### Analyses using microRNA target predicting databases

miRmap (http://mirmap.ezlab.org/) is an open-source software library which can provide comprehensive prediction of microRNA targets 24. The putative target genes could be identified by calculating the complementary ability of microRNA-mRNA interactions. The prediction results provide a list of putative target genes with miRmap scores, which are predictive reference values representing the repression strength of the microRNAs on a target mRNA. In this study, the criteria for selection of putative microRNA targets were miRmap score ≥ 97.0.

TargetScan (http://www.targetscan.org) is an online database predicting the target of microRNA by searching for the presence of conserved 8mer, 7mer, and 6mer sites matching the seed region of each microRNA 25. The results of predictions are ranked by the predicted efficacy of targeting or by their probability of conserved targeting 25. TargetScan could provide a valuable resource for investigating the role of microRNAs in gene-regulatory networks.

miRDB (http://mirdb.org) provides web-based microRNA-target prediction and functional annotations in five species, including human, mouse, rat, dog, and chicken 26, 27. In miRDB, all targets were predicted by MirTarget, which was developed by analyzing microRNA-target interactions from high-throughput sequencing experiments.

### Analysis using STRING

The functional interactions between expressed proteins in cells are very important and complicated. STRING database (https://string-db.org/) has collected and integrated this information, by consolidating known and predicted protein-protein association data of various organisms 28. The protein-protein interactions, including direct (physical) and indirect (functional) interactions, collected in STRING are derived from five main sources, including conserved co-expressions, high-throughput lab experiment, genomic context predictions, automated text-mining, and previous knowledge in database. In this study, the significantly dysregulated genes were input into STRING for protein-protein interaction network analysis. The minimum required interaction score was set to the medium confidence (score = 0.400). In addition, STRING also provides information of Kyoto Encyclopedia of Genes and Genomes (KEGG) pathway.

### Analysis using DAVID

DAVID (https://david.ncifcrf.gov/) is a powerful tool for functional classification of genes 29. It integrates gene ontology, biological process, and KEGG pathway. In DAVID database, a list of interesting genes can be classified into clusters of related biological functions, signaling pathways, or diseases by calculating the similarity of global annotation profiles with an agglomeration algorithm method. An Expression Analysis Systematic Explorer (EASE) score is a modified Fisher's exact *p* value in DAVID database which represents how specifically the genes are involved in a category. In this study, we selected EASE score = 0.1 as the default and defined pathways with a *q* value (*p* value adjusted with false discovery rate using the method by Benjamini, *et al.*) <0.05 as significant.

### Analysis using IPA

IPA (Ingenuity systems, Redwood City, CA, USA) is a database software containing large database with detailed and structured findings reviewed by experts, which was derived from thousands of biological, chemical and medical researches 30. IPA enables rapid searching, analysis, integration, and recognition of data from gene and single nucleotide polymorphism (SNP) arrays, RNA and small RNA sequencing, proteomics and many other biological experiments 30. Deeper understanding and identification of related signaling pathways, upstream regulators, molecular interactions, disease process, and candidate biomarkers are also available 30. In this study, we used IPA to assess the diseases and functions associated with the significantly dysregulated genes in endometrial adenocarcinoma. The disease and function with a *p* value < 0.05 was considered significant.

### Analysis using the Human Protein Atlas

The Human Protein Atlas is a Swedish-based program initiated in 2003 with the aim to map all the human proteins in cells, tissues, and organs using integration of various omics technologies, including antibody-based imaging, mass spectrometry-based proteomics, transcriptomics, and systems biology 31. All data collected in it is open-access to allow scientists, either in academia or industry, to freely access the data for exploration of the human proteome. The Human Protein Atlas consists of three separate parts, each focusing on a particular aspect of the genome-wide analysis of the human proteins, including the Tissue Atlas showing the distribution of the proteins across all major tissues and organs in the human body, the Cell Atlas showing the subcellular localization of proteins in single cells, and the Pathology Atlas showing the impact of protein levels for survival of cancer patients. This program has already contributed to several thousands of publications in the field of human biology and disease and it is selected by the organization ELIXIR (http://www.elixir-europe.org) as a European core resource due to its fundamental importance for a wider life science community. In this study, the prognosis-predicting values of the significantly dysregulated genes in endometrial cancer were assessed with the Human Protein Atlas. Based on the FPKM value of each gene, the patients were classified into two groups, low-expression and high-expression, to compare their prognoses. The prognosis (survival) of each group of patients was examined and compared with Kaplan-Meier survival estimators and log-rank tests. To determine the best cut-off FPKM values for grouping the patients, all FPKM values from the 20^th^ to 80^th^ percentiles were used to group the patients, and the cut-off FPKM value that yielded the lowest log-rank *p* value was selected.

## Results

### Differential gene expressions in endometrial cancer

**Figure 2:**
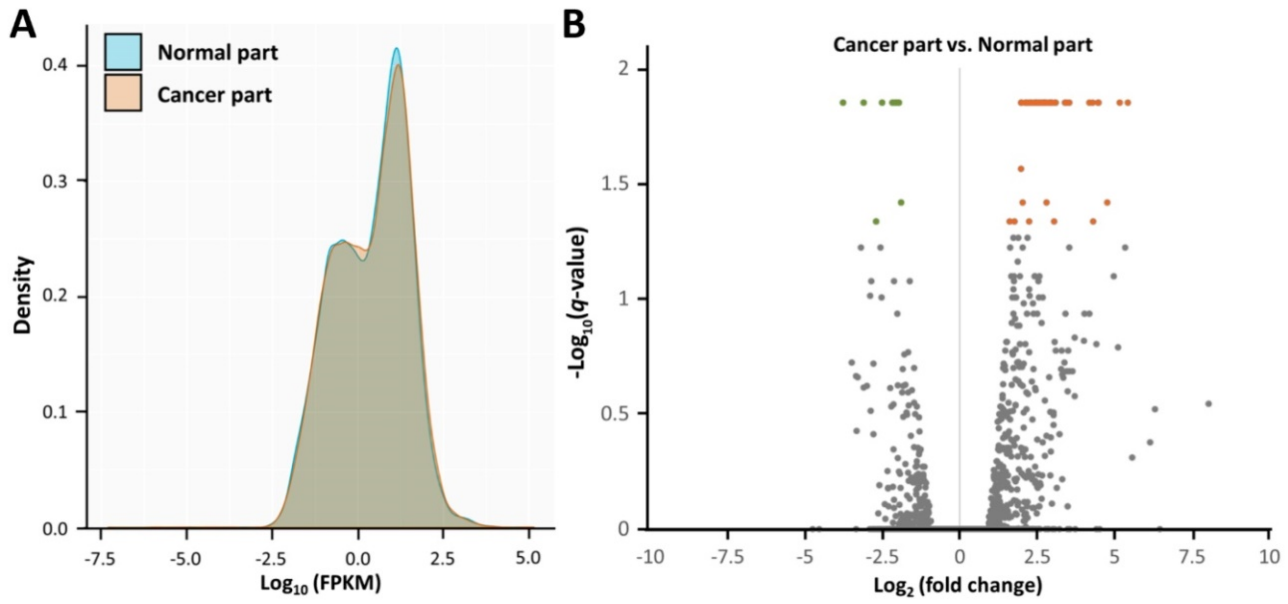
** Overview of the gene expression profiles in endometrial adenocarcinoma.** (A) The density plot illustrates smoothed frequency distribution of the fragments per kilobase of transcript per million mapped reads (FPKM) among the cancer part and non-cancer part. (B) The volcano plot of differential gene expression patterns of the cancer part vs. non-cancer part. Significantly dysregulated genes in endometrial adenocarcinoma (cancer part vs. non-cancer part) (those with -log_10_(*q*-value) > 1.3 and fold change > 2) were shown in green (downregulated) or orange (upregulated).

Using NGS, the gene expression profiles of the cancer part and non-cancer part of the surgical specimen from the patient with endometrial adenocarcinoma were assessed (Figure 2A). As shown in the volcano plots (Figure 2B), significantly dysregulated genes in endometrial adenocarcinoma (cancer part vs. non-cancer part) (those with -log_10_(*q* value) > 1.3 and fold change > 2) were identified (Table 1), including 47 upregulated and 9 downregulated genes.

**Figure 3:**
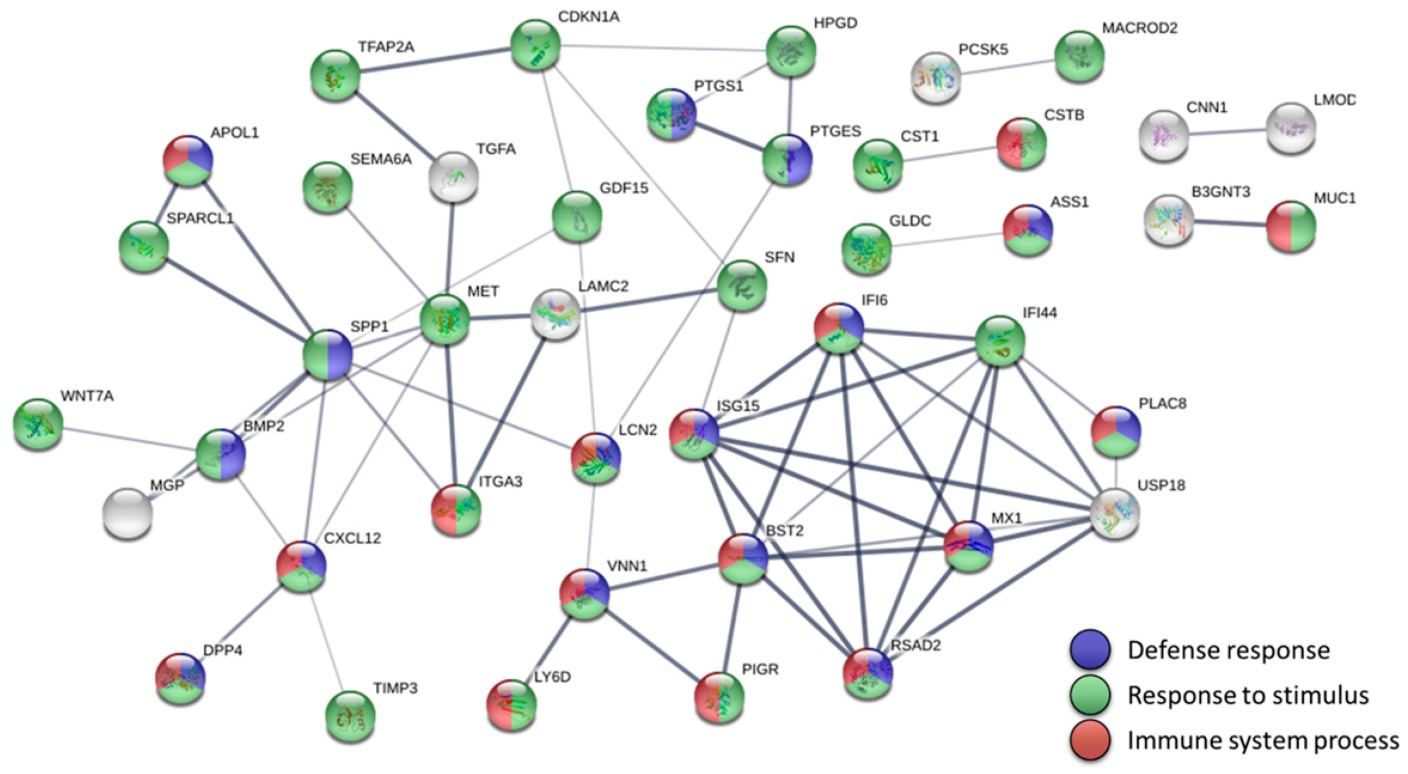
** Protein-protein interaction network analysis of the dysregulated genes in endometrial adenocarcinoma.** The 56 significantly dysregulated genes (47 upregulated and 9 downregulated) were input into the Search Tool for the Retrieval of Interacting Genes (STRING) database for protein-protein interaction (PPI) network analysis. The minimum required interaction score was set to the medium confidence (score = 0.400). Nodes represent proteins and edges represent protein-protein associations. Nodes without edges are not displayed. This analysis obtained a highly interactive PPI network of 56 nodes and 67 edges, with PPI enrichment *p* value of < 1.0 х 10^-16^. Most genes in the PPI network were associated with three biological pathways, including defense response (19 genes, shown in blue), response to stimulus (44 genes, shown in green), and immune system process (21 genes, shown in red).

Using STRING to investigate the protein-protein interactions of the significantly dysregulated genes in endometrial adenocarcinoma, we built a highly interactive protein-protein interaction (PPI) network of 56 nodes and 67 edges (enrichment *p* value < 1.0 x 10^-16^) (Figure 3). Most genes in the PPI network were associated with three biological pathways, including defense response (19 genes), response to stimulus (44 genes), and immune system process (21 genes). Furthermore, the KEGG pathway analysis indicated that human papillomavirus (HPV) infection might be the most significant pathway involved in endometrial adenocarcinoma (*q* value = 0.0038) (Table 2).

We then used DAVID to analyze the biological processes, cellular components, and molecular functions associated with the 56 significantly dysregulated genes in endometrial adenocarcinoma (Table 3). The significant biological processes included response to virus (6 genes) and type I interferon signaling pathway (5 genes). The significant cellular components included extracellular space (19 genes), extracellular exosome (25 genes), and cell surface (9 genes). The only significant molecular functions associated with the 56 significantly dysregulated genes was protease binging (7 genes).

**Figure 4:**
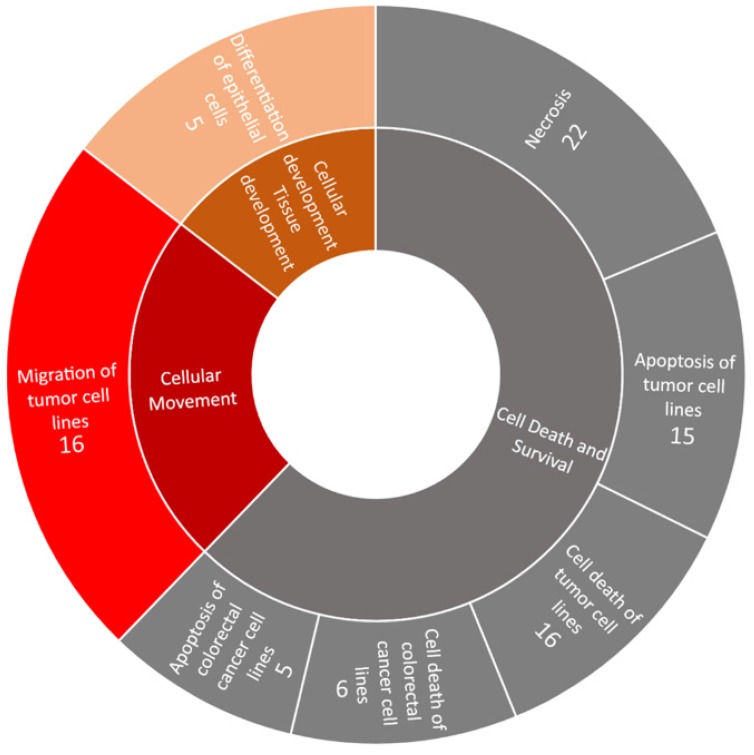
** Disease and function analysis of the significantly dysregulated genes in endometrial adenocarcinoma.** Using Ingenuity^®^ Pathway Analysis, the associated diseases and functions of the significantly dysregulated genes in endometrial adenocarcinoma were analyzed. Significant diseases and functions (those with a *p* value < 0.05) are shown in the outer circle, while their categories are shown in the inner circle. The numbers show the counts of involved genes. The area of each disease and function reflects its significant level, based on their -log_10_(*p*-value). Downregulated diseases and functions are shown in gray color, while upregulated ones are shown in orange and red colors.

Using IPA, the associated diseases and functions of the 56 significantly dysregulated genes in endometrial adenocarcinoma were investigated (Figure 4). The diseases and functions significantly associated with these dysregulated genes belonged to three categories, including cell death and survival (downregulated), cellular movement (upregulated), and cellular development and tissue development (upregulated).

### The possible genes associated with prognosis of endometrial cancer

**Figure 5:**
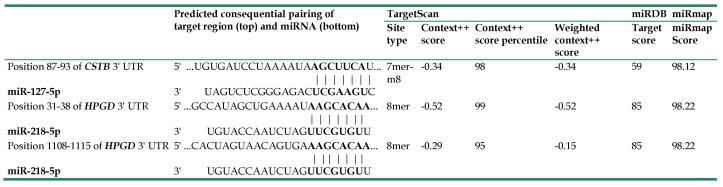

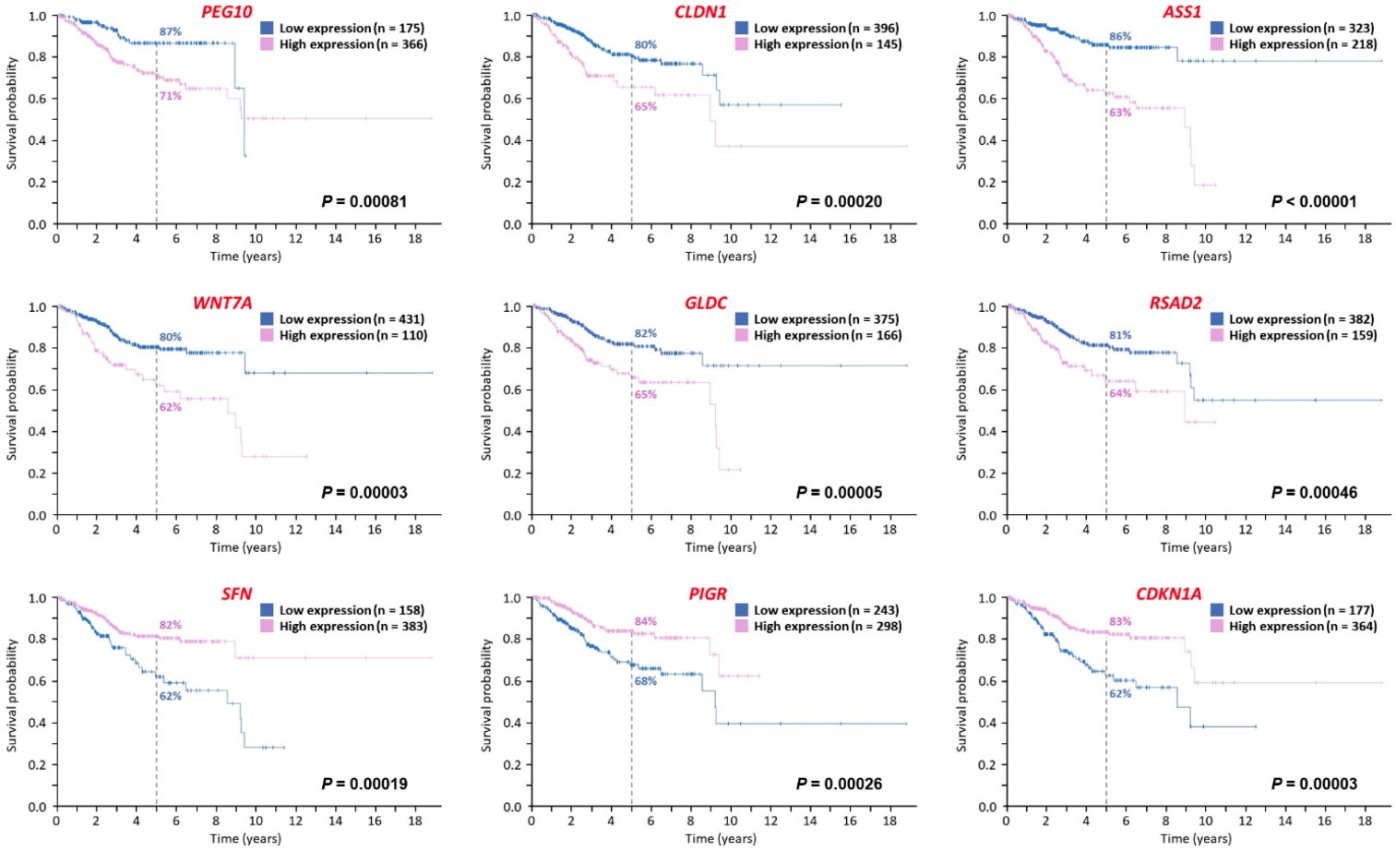
** Possible genes associated with the prognosis of endometrial cancer as predicted by the Human Protein Atlas.** The prognosis-predicting values of the 56 significantly dysregulated genes in endometrial cancer were assessed using the Human Protein Atlas. The 12 Kaplan-Meier curves (with log-rank *p* values) show the genes significantly associated with prognosis, including 6 genes associated with poorer prognosis and 3 genes associated with better prognosis.

Using the Human Protein Atlas, the prognosis-predicting values of the 56 significantly dysregulated genes in endometrial cancer were assessed (Figure 5). Totally, the information of 541 patients with endometrial cancer (Table 4) was obtained for analyses. The median follow-up time of this cohort was 2.5 years. Among these 56 significantly dysregulated genes, 6 genes were significantly associated with poorer prognosis (*PEG10*, *CLDN1*, *ASS1*, *WNT7A*, *GLDC*, and *RSAD2*) and 3 genes were significantly associated with better prognosis (*SFN*, *PIGR*, and *CDKN1A*).

### Potential dysregulated microRNA-mRNA interactions in endometrial cancer

Using NGS, 227 significantly dysregulated microRNAs (>2 fold change, including 186 upregulated and 41 downregulated microRNAs) were identified. We predicted the potential targets of these microRNAs with miRmap database, selecting the targets in the list of the 56 significantly dysregulated genes in endometrial cancer and the *microRNA-mRNA interactions with* miRmap score ≥ 97.0, and found 34 possible microRNA-mRNA interactions (including 12 interactions between a downregulated microRNA and an upregulated mRNA and 22 interactions between an upregulated microRNA and a downregulated mRNA) involving 16 mRNAs (9 upregulated and 7 downregulated mRNAs) (Figure 1). Further investigation using TargetScan and miRDB databases showed that only two microRNA-mRNA interactions, downregulated hsa-miR-127-5p with upregulated *CSTB* and upregulated hsa-miR-218-5p with downregulated *HPGD*, were validated in both TargetScan and miRDB databases (Table 5).

## Discussion

In the current study, significantly dysregulated genes, especially those mediated by dysregulated microRNAs, in endometrial adenocarcinoma were investigated comprehensively using an approach with NGS and bioinformatics. We found 56 significantly dysregulated genes, including 47 upregulated and 9 downregulated genes. Most of these genes were associated with defense response, response to stimulus, and immune system process, suggesting the important association between endometrial adenocarcinoma and immune response. Interestingly, the KEGG pathway analysis in STRING showed that HPV infection was the most significant pathway in endometrial adenocarcinoma, suggesting the possible role of HPV in the carcinogenesis of endometrial cancer. Further analyses using DAVID also implied that endometrial adenocarcinoma might be associated with virus infection. On the other hand, the analyses using IPA found that these 56 genes were associated with decreased cell death and survival as well as increased cellular movement, which were common behaviors of malignant cells. The analyses using Human Protein Atlas, identified 6 genes significantly associated with poorer prognosis and 3 genes significantly associated with better prognosis. Combining with the data of microRNA profiles using microRNA target predicting tools, two significantly dysregulated microRNA-mediated gene expression changes in endometrial adenocarcinoma were found: downregulated hsa-miR-127-5p with upregulated *CSTB* and upregulated hsa-miR-218-5p with downregulated *HPGD*.

It has been debated whether endometrial cancer is associated HPV, although a strong association between HPV and cervical cancer is well-known. The close anatomical proximity to the cervix has led researchers to study whether HPV has a role in the carcinogenesis of endometrial cancer. However, a systematic review and meta-analysis revealed a pooled prevalence of HPV DNA in endometrial cancer tissue of 10.0% (95% confidence interval: 5.2-16.2%) with large between-study heterogeneity related to the HPV DNA detection methods, and concluded that HPV had a limited or no role in the carcinogenesis of endometrial cancer 2. Nevertheless, the absence of HPV DNA in the endometrial cancer tissue cannot totally exclude the possible contribution of HPV in the pathogenesis of endometrial cancer. In our study, however, the significantly dysregulated genes in endometrial adenocarcinoma was associated with immune responses, and the KEGG pathway analysis showed that HPV infection was the most significant pathway, suggesting the possible role of HPV in the carcinogenesis of endometrial cancer. Further large-scale study is needed to elucidate the association between HPV and endometrial cancer.

Aberrant expression of various microRNAs in endometrial cancer has been reported 32. Significantly higher serum levels of miR-186, miR-222, and miR-223 and significantly lower serum level of miR-204 were noted in patients of endometrial carcinoma than in the matched control subjects 33. Some studies also demonstrated the possibility of using extracellular vesicles isolated from the peritoneal lavage as biomarkers of endometrial cancer 34. In our study, we found significantly downregulated has-miR-127-5p and upregulated has-miR-218-5p in endometrial adenocarcinoma. In line with our findings, Dong *et al.* have also demonstrated downregulation of miR-127 in endometrial cancer using microRNA microarray profiling 35 and Delangle *et al.* have shown upregulation of miR-218 in endometrial cancer tissue using quantitative reverse transcription polymerase chain reaction 32, 36.

The *CSTB* gene encodes cystatin B in humans, which may interact with cathepsin B 37. Cysteine cathepsins are highly upregulated in many cancers 38. Being in various locations (being secreted, cell-surface, and intracellular space), they involve in many proteolytic pathways that may contribute to the progression of cancers 38. In human epithelial ovarian tumors, cystatin B was a progression marker, which was associated with the transforming growth factor β (TGF-β) signaling pathway 39. In a previous study enrolling 27 patients of endometrial cancer, increased expression of cathepsin B was found as a predictor of more aggressive cancer behavior over time, suggesting its potential as being a tumor marker of unfavorable outcome 40. In the present study, significantly increased *CSTB* expression was noted in the cancer tissue, which might be related to the early stage of the cancer in our patient. Further study should be taken to elucidate the role of CSTB.

The *HPGD* gene encodes 15-hydroxyprostaglandin dehydrogenase, an important enzyme responsible for inactivating prostaglandins and associated eicosanoids via reducing the 15S-hydroxyl group, which have been demonstrated as an important factor of disease-associated pain and inflammation in patients with endometriosis 41. Decreased *HPGD* expression is associated with abnormal prostaglandin metabolism in endometriosis 42. Generally speaking, 15-hydroxyprostaglandin dehydrogenase is considered a tumor suppressor 43. It has been reported that *HPGD* is associated with various types of cancer, such as bladder cancer, gastrointestinal cancer, breast cancer, and cervical cancer 44-47. In a recent study, decreased inactivation of prostaglandins E2 and F2α (PGE_2_ and PGF_2α_) via 15-hydroxyprostaglandin dehydrogenase was noted in type II endometrial cancer, while low *HPGD* expression was associated with worse progression-free survival and overall survival 48. In line with the previous studies, we found decreased *HPGD* expression in endometrial adenocarcinoma tissue, which might be suppressed by upregulated miR-218-5p.

A major limitation of this study should be specified. This study was conducted mainly using the NGS data of a pair of tissue from a patient. Since it was mainly a single subject study, the findings might not be applied to other patients. However, our study provided insights to understand the pathogenic mechanisms of endometrial adenocarcinoma. After further validation, the potential targets identified in our study might provide scientific basis for developing novel diagnostic and treatment modalities for endometrial cancer.

In summary, we identified 56 significantly dysregulated genes in endometrial adenocarcinoma. These genes were involved in defense response, response to stimulus, and immune system process, as well as the pathway associated with HPV infection. In these genes, 6 genes were associated with poorer prognosis and 3 genes were associated with better prognosis. We further found two significantly dysregulated microRNA-mediated gene expression alterations in endometrial adenocarcinoma: downregulated hsa-miR-127-5p with upregulated *CSTB* and upregulated hsa-miR-218-5p with downregulated *HPGD*. These findings may contribute important new insights into possible novel diagnostic or therapeutic strategies for endometrial cancer.

## Figures and Tables

**Table 1:** Differentially expressed genes in endometrial adenocarcinoma (cancer part versus non-cancer part).

Official gene symbol	FPKM		Ratio (C/N)	Log_2_(ratio)	*p* value	*q* value^*^
	Cancer (C) part	Non-cancer (N) part				
***DKK4***	169.43	3.95	42.84	5.42	<0.0001	0.0141
***RXFP1***	97.99	2.73	35.86	5.16	<0.0001	0.0141
***LY6D***	41.57	1.54	27.05	4.76	0.0002	0.0384
***DPP4***	165.45	7.38	22.41	4.49	<0.0001	0.0141
***CST1***	122.75	5.56	22.07	4.46	<0.0001	0.0141
***BMP2***	18.22	0.92	19.80	4.31	0.0002	0.0462
***PTGES***	51.92	2.67	19.41	4.28	<0.0001	0.0141
***MUC13***	28.01	1.55	18.08	4.18	<0.0001	0.0141
***VNN1***	36.31	3.14	11.56	3.53	<0.0001	0.0141
***TFAP2A***	8.60	0.79	10.89	3.45	<0.0001	0.0141
***MACROD2***	39.50	3.77	10.47	3.39	<0.0001	0.0141
***SFN***	145.86	17.02	8.57	3.10	<0.0001	0.0141
***GPRC5A***	61.80	7.33	8.43	3.08	<0.0001	0.0141
***PCSK5***	9.55	1.15	8.29	3.05	0.0002	0.0462
***PEG10***	21.40	2.71	7.89	2.98	<0.0001	0.0141
***GLDC***	16.03	2.07	7.74	2.95	<0.0001	0.0141
***ISG15***	316.92	42.11	7.53	2.91	<0.0001	0.0141
***ITGA3***	76.72	10.39	7.39	2.88	<0.0001	0.0141
***LAMC2***	97.12	13.89	6.99	2.81	<0.0001	0.0141
***BATF2***	37.82	5.46	6.93	2.79	0.0002	0.0384
***PIGR***	47.74	7.12	6.70	2.74	<0.0001	0.0141
***CLDN1***	93.32	14.02	6.66	2.74	<0.0001	0.0141
***RHOF***	45.21	6.79	6.65	2.73	<0.0001	0.0141
***SEMA6A***	9.58	1.44	6.65	2.73	<0.0001	0.0141
***IFI6***	488.10	74.30	6.57	2.72	<0.0001	0.0141
***APOL1***	259.11	40.36	6.42	2.68	<0.0001	0.0141
***LCN2***	165.99	27.42	6.05	2.60	<0.0001	0.0141
***B3GNT3***	26.10	4.40	5.94	2.57	<0.0001	0.0141
***GPX3***	77.86	13.69	5.69	2.51	<0.0001	0.0141
***ASS1***	88.14	15.91	5.54	2.47	<0.0001	0.0141
***SPP1***	1680.24	304.79	5.51	2.46	<0.0001	0.0141
***F3***	151.88	27.62	5.50	2.46	<0.0001	0.0141
***RSAD2***	97.04	17.65	5.50	2.46	<0.0001	0.0141
***PLAC8***	80.96	14.73	5.49	2.46	<0.0001	0.0141
***TGFA***	27.11	5.13	5.28	2.40	<0.0001	0.0141
***CSTB***	152.48	30.76	4.96	2.31	<0.0001	0.0141
***WNT7A***	57.35	11.73	4.89	2.29	<0.0001	0.0141
***USP18***	33.36	7.11	4.69	2.23	0.0002	0.0462
***MX1***	73.00	15.58	4.69	2.23	<0.0001	0.0141
***GDA***	99.40	22.50	4.42	2.14	<0.0001	0.0141
***GDF15***	71.56	16.45	4.35	2.12	<0.0001	0.0141
***IFI44***	277.77	67.96	4.09	2.03	0.0002	0.0384
***BST2***	302.60	75.82	3.99	2.00	<0.0001	0.0141
***PTGS1***	44.54	11.28	3.95	1.98	0.0001	0.0274
***ATP11A***	24.91	6.32	3.94	1.98	<0.0001	0.0141
***CDKN1A***	106.16	31.41	3.38	1.76	0.0002	0.0462
***MET***	78.65	25.94	3.03	1.60	0.0002	0.0462
***CXCL12***	20.82	77.22	0.27	-1.89	0.0002	0.0384
***MGP***	195.18	766.67	0.25	-1.97	<0.0001	0.0141
***SPARCL1***	71.61	295.44	0.24	-2.04	<0.0001	0.0141
***TIMP3***	7.22	30.83	0.23	-2.09	<0.0001	0.0141
***HPGD***	175.32	789.89	0.22	-2.17	<0.0001	0.0141
***LMOD1***	2.55	14.59	0.17	-2.52	<0.0001	0.0141
***PDLIM3***	3.18	20.66	0.15	-2.70	0.0002	0.0462
***CNN1***	2.98	25.60	0.12	-3.10	<0.0001	0.0141
***DES***	4.10	55.87	0.07	-3.77	<0.0001	0.0141

* *p* values adjusted with false discovery rate. Abbreviation: FPKM, fragments per kilobase of transcript per million mapped reads. The genes highlighted with underlines were those significantly associated with poorer (in red color) or better (in blue color) prognosis, as shown in Figure 5.

**Table 2:** KEGG pathway analysis of the significantly dysregulated genes in endometrial adenocarcinoma using the STRING database.

Term description	Observed gene count	*q* value^*^	Matching proteins in the network
Human papillomavirus infection	7	0.0038	*CDKN1A*, *ISG15*, *ITGA3*, *LAMC2*, *MX1*, *SPP1*, *WNT7A*
Pathways in cancer	8	0.0058	*BMP2*, *CDKN1A*, *CXCL12*, *ITGA3*, *LAMC2*, *MET*, *TGFA*, *WNT7A*
PI3K-Akt signaling pathway	6	0.0176	*CDKN1A*, *ITGA3*, *LAMC2*, *MET*, *SPP1*, *TGFA*
Arachidonic acid metabolism	3	0.0213	*GPX3*, *PTGES*, *PTGS1*
Renal cell carcinoma	3	0.0213	*CDKN1A*, *MET*, *TGFA*
Basal cell carcinoma	3	0.0213	*BMP2*, *CDKN1A*, *WNT7A*
Hepatocellular carcinoma	4	0.0213	*CDKN1A*, *MET*, *TGFA*, *WNT7A*
ECM-receptor interaction	3	0.0234	*ITGA3*, *LAMC2*, *SPP1*
Focal adhesion	4	0.0291	*ITGA3*, *LAMC2*, *MET*, *SPP1*
Proteoglycans in cancer	4	0.0291	*CDKN1A*, *MET*, *TIMP3*, *WNT7A*
Small cell lung cancer	3	0.0291	*CDKN1A*, *ITGA3*, *LAMC2*

* *p* values adjusted with false discovery rate. The genes highlighted with underlines were those significantly associated with poorer (in red color) or better (in blue color) prognosis, as shown in Figure 5. Abbreviation: KEGG, Kyoto Encyclopedia of Genes and Genomes; STRING, Search Tool for the Retrieval of Interacting Genes.

**Table 3:** The significant biological processes (BP), cellular components (CC), and molecular function (MF) of the significantly dysregulated genes in endometrial adenocarcinoma shown in DAVID.^a^

Category	Term	Gene count	Genes	*q* value^*^
BP	Response to virus	6	*LCN2*, *BST2*, *RSAD2*, *IFI44*, *MX1*, *CXCL12*	0.016
BP	Type I interferon signaling pathway	5	*ISG15*, *BST2*, *RSAD2*, *MX1*, *IFI6*	0.015
CC	Extracellular space	19	*BMP2*, *SPARCL1*, *CST1*, *SFN*, *PIGR*, *TIMP3*, *CXCL12*, *LCN2*, *APOL1*, *F3*, *GPX3*, *CSTB*, *TGFA*, *LAMC2*, *GDF15*, *WNT7A*, *PCSK5*, *MUC13*, *SPP1*	<0.001
CC	Extracellular exosome	25	*GDA*, *ASS1*, *BST2*, *SPARCL1*, *PTGS1*, *MGP*, *ITGA3*, *SFN*, *PIGR*, *GPRC5A*, *TIMP3*, *CXCL12*, *LCN2*, *DES*, *F3*, *GPX3*, *CSTB*, *VNN1*, *GDF15*, *HPGD*, *RHOF*, *WNT7A*, *MUC13*, *DPP4*, *SPP1*	<0.001
CC	Cell surface	9	*BMP2*, *LY6D*, *BST2*, *F3*, *MET*, *TGFA*, *ITGA3*, *WNT7A*, *DPP4*	0.006
MF	Protease binding	7	*LCN2*, *F3*, *CSTB*, *CST1*, *ITGA3*, *TIMP3*, *DPP4*	<0.001

^a^ *q* value < 0.05 was considered significant. * *p* value adjusted with false discovery rate. The genes highlighted with underlines were those significantly associated with poorer (in red color) or better (in blue color) prognosis, as shown in Figure 5. Abbreviation: DAVID, Database for Annotation, Visualization and Integrated Discovery.

**Table 4:** Baseline demographics of the endometrial cancer patients obtained from Human Protein Atlas.

Variable	Data
**Number**	541
**Age (year)** - mean (± standard deviation) ^†^	64 (±11)
**Race** - n (%)	
** American Indian or Alaska native**	4 (1%)
** Asian**	20 (4%)
** African American**	106 (20%)
** Native Hawaiian or other Pacific islander**	9 (2%)
** White**	370 (68%)
** Unknown**	32 (6%)

^†^ Ages of 3 subjects were unavailable.

**Table 5:** Potential miRNA-mRNA interactions involved in endometrial adenocarcinoma, validated with TargetScan, miRDB, and miRmap.
